# On a ratio monotonicity conjecture of a new kind of numbers

**DOI:** 10.1186/s13660-018-1614-1

**Published:** 2018-01-25

**Authors:** Brian Yi Sun

**Affiliations:** College of Mathematics and System Science, Xinjiang University, Urumqi, P.R. China

**Keywords:** 05A20, 05A10, 11B65, 11B37, log-concavity, log-convexity, ratio monotonicity, interlacing method

## Abstract

It is known that the concept of ratio monotonicity is closely related to log-convexity and log-concavity. In this paper, by exploring the log-behavior properties of a new combinatorial sequence defined by Z.-W. Sun, we completely solve a conjecture on ratio monotonicity by him.

## Introduction

To be self-contained in this paper, let us first review some necessary and important concepts.

Let $\{z_{n}\}_{n\geq0}$ be a number-theoretic or combinatorial sequence of positive numbers. It is called *(strictly) ratio monotonic* if the sequence $\{z_{n}/z_{n-1}\}_{n\geq1}$ is (strictly) monotonically increasing or decreasing. The concept of ratio monotonicity is closely related log-convexity and log-concavity. A sequence $\{z_{n}\}_{n=0}^{\infty}$ is called *log-convex (resp. log-concave)* if for all $n\geq1$,

1.1 $$ z_{n-1}z_{n+1}\geq z_{n}^{2} \quad\bigl(\text{resp.} z_{n-1}z_{n+1}\leq z_{n}^{2} \bigr). $$

Correspondingly, if the inequality in (1.1) is strict, then we call the sequence $\{z_{n}\}_{n=0}^{\infty}$ *strictly log-convex (resp. log-concave)*.

Clearly, a sequence $\{z_{n}\}_{n=0}^{\infty}$ is (strictly) log-convex (resp. log-concave) if and only if the sequence $\{z_{n+1}/z_{n}\} _{n\geq0}$ is (strictly) increasing (resp. decreasing). So, to study the ratio monotonicity is equivalent to study the log-convexity and log-concavity; see [1].

In recent years, Sun [2, 3] posed a series of conjectures on monotonicity of sequences of the forms $\{z_{n+1}/z_{n}\}_{n\geq0}^{\infty}$, $\{\sqrt[n]{z_{n}}\}_{n\geq1}$, and $\{\sqrt[n+1]{z_{n+1}}/\sqrt [n]{z_{n}}\}_{n\geq1}$. It is worth mentioning that many scholars have made valuable progress on this topic, such as Chen et al. [4], Hou et al. [5], Luca and Stănică [6], Wang an Zhu [1], Sun et al. [7], and Zhao [8].

Sun [2] posed a conjecture on ratio monotonicity of the sequence

1.2 $$ R_{n}=\sum_{k=0}^{n} \binom{n}{k} \binom{n+k}{k}\frac{1}{2k-1},\quad n=0,1,2,\ldots. $$

He conjectured that the sequence $\{R_{n}\}_{n=0}^{\infty}$ is strictly ratio increasing to the limit $3+2\sqrt{2}$ and that the sequence $\{ \sqrt[n]{R_{n}}\}_{n=1}^{\infty}$ is strictly ratio decreasing to the limit 1.

It is worth noting that Sun [2] also put forward a similar conjecture on the ratio monotonicity of the sequence

1.3 $$ S_{n}=\sum_{k=0}^{n} \binom{n}{k}^{2} \binom{2k}{k}(2k+1),\quad n=0,1,2,\ldots. $$

By using a result on the sequence $\{S_{n}\}_{n=0}^{\infty}$ in [9], Sun et al. [7] deduced a three-term recurrence for $S_{n}$ and thus completely solved this conjecture on $\{S_{n}\}_{n=0}^{\infty}$.

However, the ratio monotonicity conjecture on the sequence $\{R_{n}\} _{n=0}^{\infty}$ can not be attacked with the methods of Sun et al. [7] since there exists no three-term recurrence for $R_{n}$. In fact, we can easily acquire a four-term recurrence for $R_{n}$. For example, using the holonomic method in [10] or the Zeilberger algorithm [11, 12], we can find the following recurrence:

1.4 $$ (n+3) R_{n+3}-(7 n+13) R_{n+2}+(7 n+15) R_{n+1}-(n+1)R_{n}=0. $$

In this paper, by studying the log-behavior properties of the sequence $\{R_{n}\}_{n=0}^{\infty}$ we completely solve the ratio monotonicity conjecture on $\{R_{n}\}_{n=0}^{\infty}$.

### Theorem 1.1

*The sequence* $\{R_{n+1}/R_{n}\} _{n=3}^{\infty}$ *is strictly increasing to the limit* $3+2\sqrt{2}$, *and the sequence* $\{\sqrt[n+1]{R_{n+1}}/\sqrt[n]{R_{n}}\}_{n=5}^{\infty}$ *is strictly decreasing to the limit* 1.

In what follows, in Section 2 we first introduce the interlacing method which can be used to verify log-behavior property of a sequence. In Section 3 we establish a lower bound and an upper bound for $R_{n+1}/R_{n}$. We will give and prove some limits and log-behavior properties related to the sequence $\{R_{n}\}_{n=0}^{\infty}$ in Section 4 and finally prove Theorem 1.1 therein. In the end, we conclude this paper with some open conjectures for further research.

## The interlacing method

The interlacing method can be found in [13], yet it was formally considered as a method to solve logarithmic behavior of combinatorial sequences by Dos̆lić and Veljan [14], in which it was also called the sandwich method.

Let us give a simple introduction to this method to be self-contained in our paper. Suppose that $\{z_{n}\}_{n\geq0}^{\infty}$ is a sequence of positive numbers and let

$$q_{n}=\frac{z_{n}}{z_{n-1}},\quad n\geq1. $$

By the inequality in (1.1), the log-convexity or log-concavity of a sequence $\{z_{n}\}_{n\geq0}$ is equivalent, respectively, to $q_{n}\leq q_{n+1}$ or $q_{n}\geq q_{n+1}$ for all $n\geq1$. Generally, it is not easy to prove the monotonicity of $\{q_{n}\}_{n\geq 1}$, yet if we can find an increasing (resp. a decreasing) sequence $\{b_{n}\}_{n\geq0}$ such that

2.1 $$ b_{n-1}\leq q_{n}\leq b_{n} \quad( \text{resp. }b_{n-1}\geq q_{n}\geq b_{n}) $$

for all $n\geq1$, or at least for all $n\geq N$ for some positive integer *N*, then we can show its monotonicity. Based on these arguments, the following proposition is obvious.

### Proposition 2.1

*Suppose that* $\{z_{n}\}_{n\geq0}$ *is a sequence of positive numbers*. *Then for some positive integer* *N*, *the sequence* $\{z_{n}\}_{n\geq N}$ *is log*-*convex* (*resp*. *log*-*concave*) *if there exists an increasing*(*resp*. *a decreasing*) *sequence* $\{b_{n}\}_{n\geq0}$ *such that*

$$ b_{n-1}\leq q_{n}\leq b_{n} \quad(\textit{resp. }b_{n-1} \geq q_{n}\geq b_{n}) $$

*for* $n\geq N+1$.

## Bounds for $R_{n+1}/R_{n}$

In this section, we establish lower and upper bounds for $R_{n+1}/R_{n}$.

### Lemma 3.1

*Let* $r_{n}=\frac{R_{n+1}}{R_{n}}$ *and*

$$\begin{aligned} b_{n}&=3+2\sqrt{2}-\frac{3 (41\sqrt{2}+58)}{(14 \sqrt{2}+20) n} \\ &= \biggl(3-\frac{9}{2n} \biggr)+\sqrt{2} \biggl(2- \frac{3}{n} \biggr). \end{aligned}$$

*Then*, *for* $n\geq3$, *we have*

$$ b_{n}< r_{n}< b_{n+1}. $$

### Proof

The recurrence relationship (1.4) implies that

$$ \frac{R_{n+3}}{R_{n+2}}= \frac{7n+13}{n+3}-\frac{7n+15}{n+3}\frac{R_{n+1}}{R_{n+2}}+ \frac {n+1}{n+3}\frac{R_{n}}{R_{n+2}} \quad \mbox{for } n\geq0. $$

This equation can be rewritten as follows:

3.1 $$ r_{n+2}=\frac{7n+13}{n+3}- \frac{7n+15}{n+3}\cdot\frac{1}{r_{n+1}}+ \frac{n+1}{n+3}\cdot\frac {1}{r_{n}r_{n+1}}. $$

Now we proceed the proof by induction.

First, note that

$$b_{3}=\frac{3}{2}+\sqrt{2}\approx2.91421,\qquad b_{4}= \frac{5}{8} (3+2 \sqrt {2} )\approx3.64277,\qquad r_{3}= \frac{87}{25}\approx3.48, $$

so it is easy to verify that $b_{3}< r_{3}< b_{4}$.

Suppose that $b_{n}< r_{n}< b_{n+1}$ for $n\leq k+1$. It suffices to show that $r_{k+2}< b_{k+3}$ and $r_{k+2}>b_{k+2}$. We have

$$\begin{aligned} &r_{k+2}-b_{k+3} \\ &\quad=\frac{7k+13}{k+3}- \frac{7k+15}{k+3}\cdot\frac{1}{r_{k+1}}+ \frac{k+1}{k+3}\cdot\frac {1}{r_{k}r_{k+1}}-b_{k+3} \\ &\quad< \frac{7k+13}{k+3}- \frac{7k+15}{k+3}\cdot\frac{1}{b_{k+2}}+ \frac{k+1}{k+3}\cdot\frac{1}{b_{k}b_{k+1}} -b_{k+3} \\ &\quad=\frac {(7k+13)b_{k}b_{k+1}b_{k+2}-(7k+15)b_{k}b_{k+1}+(k+1)b_{k+2}-(k+3)b_{k}b_{k+1}b_{k+2}b_{k+3}}{ (k+3)b_{k}b_{k+1}b_{k+2}} \\ &\quad=\frac{3 (2 k (-4 (179+127 \sqrt{2} ) k+1110 \sqrt {2}+1573 )-844 \sqrt{2}-1197 )}{16 k (k+1) (k+2)(k+3)b_{k}b_{k+1}b_{k+2}} \\ &\quad=\frac{-24 (179+127 \sqrt{2} ) k^{2}+3 (3146+2220 \sqrt {2} ) k-3 (1197+844 \sqrt{2} )}{16 k (1 + k) (2 + k)(k+3)b_{k}b_{k+1}b_{k+2}}. \end{aligned}$$

Let $f(x)=ax^{2}+bx+c$, where $a=-24 (179+127 \sqrt{2} ) $, $b=3 (3146+2220 \sqrt{2} )$, and $c=-3 (1197+844 \sqrt {2} )$. So we obtain that $f(k)\leq f(3)=-3 (4647 + 3328 \sqrt{2})<0$ for $k\geq3$ since $-\frac {b}{2a}=\frac{3146+2220 \sqrt{2}}{16 (179+127 \sqrt{2} )}\approx1.09549$. This gives us $r_{k+2}-b_{k+3}<0$.

The proof of $r_{k+2}>b_{k+2}$ is similar, so we omit it for brevity.

According to the above analysis and the inductive argument, it follows that

$$b_{n}< r_{n}< b_{n+1} \quad \mbox{for all } n\geq3. $$

 □

### Remark 3.2

This bound was found by a lot of computer experiments. It is interesting to explore a unified method that can be used to find lower and upper bounds for the sequence $\{\frac{z_{n+1}}{z_{n}}\}_{n\geq0}$, where $\{z_{n}\}_{n\geq0}$ is a sequence satisfying a four-term recurrence.

## Log-behavior of the sequence $\{R_{n}\}_{n=0}^{\infty}$

In this section, some log-behavior and limits properties can be deduced by using Lemma 3.1.

### Theorem 4.1

*The sequence* $\{R_{n}\}_{n=4}^{\infty}$ *is strictly log*-*convex*. *Equivalently*, *the sequence* $\{R_{n+1}/R_{n}\}_{n=3}^{\infty}$ *is strictly increasing*.

### Proof

First, note that $R_{3}^{2}-R_{2}R_{4}=25^{2}-7\cdot87=16>0$. By Lemma 3.1 we have

$$ b_{n}< r_{n}=\frac{R_{n+1}}{R_{n}}< b_{n+1}< r_{n+1}< b_{n+2} \quad\mbox{for } n\geq3. $$

This gives that the sequence $\{r_{n}\}_{n=3}^{\infty}$ is strictly increasing, which implies that $\{R_{n}\}_{n=4}^{\infty}$ is log-convex by Proposition 2.1. □

Since

$$\begin{aligned} \lim_{n\rightarrow\infty}b_{n}=\lim_{n\rightarrow\infty} b_{n+1}=3+2\sqrt{2}, \end{aligned}$$

the following corollary easily follows.

### Corollary 4.2

*For the sequence* $\{R_{n}\}_{n=0}^{\infty}$, *we have*

$$ \lim_{n\rightarrow\infty}\frac{R_{n+1}}{R_{n}}=3+2\sqrt{2}. $$

### Theorem 4.3

*The sequence* $\{\sqrt[n]{R_{n}}\}_{n=1}^{\infty}$ *is strictly increasing*. *Moreover*,

4.1 $$ \lim_{n\rightarrow\infty}\sqrt[n]{R_{n}}=3+2\sqrt{2}. $$

### Proof

By Theorem 4.1 we have

$$ \frac{R_{n+1}}{R_{n}}>\frac{R_{n}}{R_{n-1}}\quad \mbox{for } n\geq3. $$

Since $R_{1}=1$, we can deduce that

4.2 $$ R_{n}=\frac{R_{2}}{R_{1}}\cdot\frac{R_{3}}{R_{2}} \biggl[ \cdot R_{1} \cdot\frac {R_{4}}{R_{3}} \cdots\frac{R_{n}}{R_{n-1}} \biggr]< R_{3} \biggl(\frac {R_{n+1}}{R_{n}} \biggr)^{n-2}\quad \mbox{for } n\geq1. $$

For $n\geq11$, we have

4.3 $$ \frac{R_{n+1}}{R_{n}}\geq\frac{R_{12}}{R_{11}} =\frac{16{,}421{,}831}{3{,}242{,}377}>5= \sqrt{R_{3}}. $$

Combining (4.2) and (4.3) gives us

$$ R_{n}^{n+1}< R_{n+1}^{n}\quad \text{for } n \geq11. $$

This is equivalent to

$$ \bigl(R_{n}^{n+1}\bigr)^{\frac{1}{n(n+1)}} < \bigl(R_{n+1}^{n} \bigr)^{\frac{1}{n(n+1)}}\quad\text{for } n\geq11, $$

that is,

$$ \sqrt[n]{R_{n}}< \sqrt[n+1]{R_{n+1}} \quad\text{for }n\geq11. $$

For $1\leq n\leq10$, we can simply prove that $R_{n}^{n+1}< R_{n+1}^{n}$ by computing the value of $R_{n}^{n+1}-R_{n+1}^{n}$. Here are some examples:

$$\begin{aligned} &R_{1}^{2}-R_{2}=1-7=-6; \\ &R_{2}^{3}-R_{3}^{2}=343-625=-282; \\ &R_{3}^{4}-R_{4}^{3}=390{,}625-658{,}503=-267{,}878; \\ &R_{4}^{5}-R_{5}^{4}=4{,}984{,}209{,}207-1{,}268{,}163{,}904{,}241{,}521=-673{,}1904{,}874; \\ &R_{5}^{6}-R_{6}^{5}= 1{,}268{,}163{,}904{,}241{,}521-1{,}268{,}163{,}904{,}241{,}521\\ &\phantom{R_{5}^{6}-R_{6}^{5}}=-3{,}367{,}343{,}548{,}629{,}278. \end{aligned}$$

Moreover, recall that, for a real sequence $\{z_{n}\}_{n=1}^{\infty}$ with positive numbers, it was shown that

4.4 $$ \lim_{n\rightarrow\infty}\inf\frac{z_{n+1}}{z_{n}}\leq\lim _{n\rightarrow \infty}\inf\sqrt[n]{z_{n}} $$

and

4.5 $$ \lim_{n\rightarrow\infty}\sup\sqrt[n]{z_{n}}\leq\lim _{n\rightarrow\infty }\sup\frac{z_{n+1}}{z_{n}}; $$

see Rudin [15, Section 3.37]. The inequalities in (4.4) and (4.5) imply that

$$\lim_{n\rightarrow\infty}\sqrt[n]{z_{n}}= \lim_{n\rightarrow\infty} \frac{z_{n}}{z_{n-1}} $$

if $\lim_{n\rightarrow\infty}\frac{z_{n}}{z_{n-1}}$ exists. Now (4.1) follows by Corollary 4.2.

This completes the proof. □

### Theorem 4.4

*For the sequence* $\{\sqrt[n]{R_{n}}\}_{n=1}^{\infty}$, *we have*

$$ \lim_{n\rightarrow\infty}\frac{\sqrt[n+1]{R_{n+1}}}{\sqrt[n]{R_{n}}} =1. $$

### Proof

Consider

$$ R_{n+1}=R_{3}\prod_{k=3}^{n}r_{k} \quad\mbox{for } n\geq3. $$

Hence, by Lemma 3.1 it follows that

$$ R_{3}\prod_{k=3}^{n}b_{k}< R_{n+1}< R_{3} \prod_{k=3}^{n}b_{k+1}. $$

We have

$$\begin{aligned} \log \biggl(\frac{\sqrt[n+1]{R_{n+1}}}{\sqrt[n]{R_{n}}} \biggr) &=\frac{\log R_{n+1}}{n+1}- \frac{\log R_{n}}{n} \\ &< \frac{\log (R_{3}\prod_{k=3}^{n}b_{k+1} )}{n+1}-\frac{\log (R_{3}\prod_{k=3}^{n-1}b_{k} )}{n} \\ &=\frac{\log R_{3}+\sum_{k=3}^{n}\log b_{k+1}}{n+1}-\frac{\log R_{3} +\sum_{k=3}^{n-1}\log b_{k}}{n} \end{aligned}$$

and

$$\begin{aligned} \log \biggl(\frac{\sqrt[n+1]{R_{n+1}}}{\sqrt[n]{R_{n}}} \biggr) &= \frac{\log R_{n+1}}{n+1}-\frac{\log \left .R_{n}\right .}{n} \\ &>\frac{\log (R_{3}\prod_{k=3}^{n}b_{k} )}{n+1}-\frac{\log (R_{3}\prod_{k=3}^{n-1}b_{k+1} )}{n} \\ &=\frac{\log R_{3}+\sum_{k=3}^{n}\log b_{k}}{n+1}-\frac{\log R_{3} +\sum_{k=3}^{n-1}\log b_{k+1}}{n}. \end{aligned}$$

Since $b_{n}$ is an increasing function with respect to *n* and positive for all $n\geq3$, we have

$$\frac{b_{n}b_{n+1}}{b_{3}}\geq\frac{b_{3}b_{4}}{b_{3}}=\frac{5}{8} (3+2 \sqrt{2} )>1. $$

This gives us that

$$\begin{aligned} \sum_{k=3}^{n}\log b_{k+1}-\sum _{k=3}^{n-1}\log b_{k}&=\log \frac {b_{n}b_{n+1}}{b_{3}}>0, \end{aligned}$$

and thus we have

4.6 $$\begin{aligned} \sum_{k=3}^{n}\log b_{k+1}>\sum_{k=3}^{n-1}\log b_{k}. \end{aligned}$$

On the one hand, using inequality (4.6), it follows that, for $n\geq3$,

$$\begin{aligned} &\frac{\log R_{3}+\sum_{k=3}^{n}\log b_{k+1}}{n+1}-\frac{\log R_{3} +\sum_{k=3}^{n-1}\log b_{k}}{n} \\ &\quad > \Biggl(\log R_{3}+\sum_{k=3}^{n} \log b_{k+1} \Biggr) \biggl(\frac {1}{n+1}-\frac{1}{n} \biggr) \\ &\quad =-\frac{\log R_{3}+\sum_{k=3}^{n}\log b_{k+1}}{n(n+1)} \\ &\quad >-\frac{\log R_{3}+(n-2)\log b_{4}}{n(n+1)}. \end{aligned}$$

On the other hand, for $n\geq3$, we can deduce that

$$\begin{aligned} &\frac{\log R_{3}+\sum_{k=3}^{n}\log b_{k+1}}{n+1}-\frac{\log R_{3} +\sum_{k=3}^{n-1}\log b_{k}}{n} \\ &\quad < \frac{\log b_{n}+\log b_{n+1}-\log b_{3}}{n} \\ & \quad < \frac{2\log b_{n+1}-\log b_{3}}{n}. \end{aligned}$$

Since $b_{n}$ is bounded, we have

$$\begin{aligned} \lim_{n\rightarrow\infty}\frac{\log R_{3}+(n-2)\log b_{4}}{n(n+1)}=0 \end{aligned}$$

and

$$\begin{aligned} \lim_{n\rightarrow\infty}\frac{2\log b_{n+1}-\log b_{3}}{n}=0, \end{aligned}$$

which implies that

4.7 $$\begin{aligned} \lim_{n\rightarrow\infty} \biggl(\frac{\log R_{3}+\sum_{k=3}^{n}\log b_{k+1}}{n+1}- \frac{\log R_{3} +\sum_{k=3}^{n-1}\log b_{k}}{n} \biggr)=0. \end{aligned}$$

Similarly, with the same argument, we can also obtain that

4.8 $$\begin{aligned} \lim_{n\rightarrow\infty} \biggl(\frac{\log R_{3}+\sum_{k=3}^{n}\log b_{k}}{n+1}- \frac{\log R_{3} +\sum_{k=3}^{n-1}\log b_{k+1}}{n} \biggr)=0. \end{aligned}$$

The limits (4.7) and (4.8) imply that

$$\lim_{n\rightarrow\infty}\log \biggl(\frac{\sqrt[n+1]{R_{n+1}}}{\sqrt [n]{R_{n}}} \biggr)=0, $$

and thus

$$\lim_{n\rightarrow\infty}\frac{\sqrt[n+1]{R_{n+1}}}{\sqrt[n]{R_{n}}}=1. $$

 □

### Theorem 4.5

*The sequence* $\{\sqrt[n]{R_{n}}\}_{n=5}^{\infty}$ *is strictly log*-*concave*. *Equivalently*, *the sequence* $\{\frac{\sqrt[n+1]{R_{n+1}}}{\sqrt [n]{R_{n}}}\}_{n=5}^{\infty}$ *is strictly decreasing*.

Before giving the proof of Theorem 4.5, we have to use to a criterion for log-concavity of sequences in the form of $\{\sqrt[n]{z_{n}}\}_{n=1}^{\infty}$; this criterion was established by Xia [16].

### Theorem 4.6

([16, Theorem 2.1]) *Let* $\{z_{n}\}_{n=0}^{\infty}$ *be a positive sequence*. *Suppose that there exist positive number* $k_{0}$, *positive integer* $N_{0}$, *and a function* $f(n)$ *such that* $k_{0}< N_{0}^{2}+N_{0}+2$ *and*, *for* $n\geq N_{0}$,

(i) $0< f(n)<\frac{z_{n}}{z_{n-1}}<f(n+1)$;
(ii) $\frac{f(n+1)}{f(n+3)}>1-\frac{k_{0}}{n^{2}+n+2}$;
(iii) $(1-\frac{k_{0}}{N_{0}^{2}+N_{0}+2} )^{N_{0}^{2}+N_{0}+2} f^{2N_{0}}(N_{0})>z_{N_{0}}^{2}$.

*Then the sequence* $\{\sqrt[n]{z_{n}}\}_{n=N_{0}}^{\infty}$ *is strictly log*-*concave*.

We are now in a position to prove Theorem 4.5.

### Proof of Theorem 4.5

Let $f(n)=b_{n-1}=\sqrt{2} (2-\frac{3}{n-1} )-\frac{9}{2 (n-1)}+3$. First, by Lemma 3.1 we have

$$ 0< f(n)< \frac{R_{n}}{R_{n-1}}< f(n+1)\quad\text{for }n\geq5. $$

Note that

$$\frac{f(n+1)}{f(n+3)}=\frac{12}{2 n+1}-\frac{6}{n}+1 $$

and

$$\begin{aligned} & \biggl(\frac{12}{2 n+1}-\frac{6}{n}+1 \biggr)- \biggl(1- \frac{4}{n^{2}+n+2} \biggr) \\ &\quad =\frac{2 (n-3) (n+2)}{n (2 n+1) (n^{2}+n+2 )} \\ &\quad >0\quad \text{for }n\geq4. \end{aligned}$$

So, taking $k_{0}=4$, condition (ii) in Theorem 4.6 is satisfied.

Moreover, note that

$$\begin{aligned} \biggl(1-\frac{4}{8^{2}+8+2} \biggr)^{8^{2}+8+2} f^{16}(8)-R_{8}^{2}=-1.5798 \times10^{8} \end{aligned}$$

and

$$\begin{aligned} \biggl(1-\frac{4}{9^{2}+9+2} \biggr)^{9^{2}+9+2} f^{18}(9)-R_{9}^{2}=6.41905 \times10^{9}. \end{aligned}$$

Therefore, with $N_{0}=9, k_{0}=4$, and $f(n)=b(n-1)$, all conditions (i), (ii), and (iii) in Theorem 4.6 are satisfied. This implies that the sequence $\{\sqrt[n]{R_{n}}\}_{n=9}^{\infty}$ is strictly log-concave, which is equivalent to that $\{\frac{\sqrt[n+1]{R_{n+1}}}{\sqrt[n]{R_{n}}}\} _{n=9}^{\infty}$ is strictly decreasing by Proposition 2.1.

However, we can verify that, for $5\leq n\leq8$,

$$\begin{aligned} \frac{\sqrt[n+1]{R_{n+1}}}{\sqrt[n]{R_{n}}}>\frac{\sqrt [n+2]{R_{n+2}}}{\sqrt[n+1]{R_{n+1}}}, \end{aligned}$$

since

$$\begin{aligned} &\frac{\sqrt[6]{R_{6}}}{\sqrt[5]{R_{5}}} -\frac{\sqrt[7]{R_{7}}}{\sqrt[6]{R_{6}}}\approx0.00293164,\qquad\frac{\sqrt[7]{R_{7}}}{\sqrt[6]{R_{6}}} - \frac{\sqrt[8]{R_{8}}}{\sqrt[7]{R_{7}}}\approx0.00445875, \\ &\frac{\sqrt[8]{R_{8}}}{\sqrt[7]{R_{7}}} -\frac{\sqrt[9]{R_{9}}}{\sqrt[8]{R_{8}}}\approx0.00452784,\qquad\frac{\sqrt[9]{R_{9}}}{\sqrt[8]{R_{8}}} - \frac{\sqrt[10]{R_{10}}}{\sqrt[9]{R_{9}}}\approx0.00404051. \end{aligned}$$

This completes the proof. □

Now we are ready to prove Theorem 1.1.

### Proof of Theorem 1.1

By Theorems 4.1 and 4.2 we confirm the first part of Theorem 1.1. Moreover, Theorems 4.5 and 4.4 imply the second part of Theorem 1.1. This ends the proof. □

We conclude the paper with some conjectures for further research.

### Conjecture 4.7

*The sequence* $\{\frac{R_{n+1}}{R_{n}}\}_{n\geq4}$ *is log*-*concave*, *that is*, $R_{n}$ *is ratio log*-*concave for* $n\geq4$.

### Conjecture 4.8

*The sequence* $\{R_{n}^{2}-R_{n+1}R_{n-1}\}_{n\geq6}$ *is* ∞-*log*-*concave*.
